# First person – Theja Abayarathna

**DOI:** 10.1242/bio.044032

**Published:** 2019-04-15

## Abstract

First Person is a series of interviews with the first authors of a selection of papers published in Biology Open, helping early-career researchers promote themselves alongside their papers. Theja Abayarathna is first author on ‘ Higher incubation temperatures produce long-lasting upward shifts in cold tolerance, but not heat tolerance, of hatchling geckos’, published in BiO. Theja is a PhD student in the lab of Associate Professor Jonathan Webb at the University of Technology Sydney, Australia, investigating the effects of climate change on geckos.


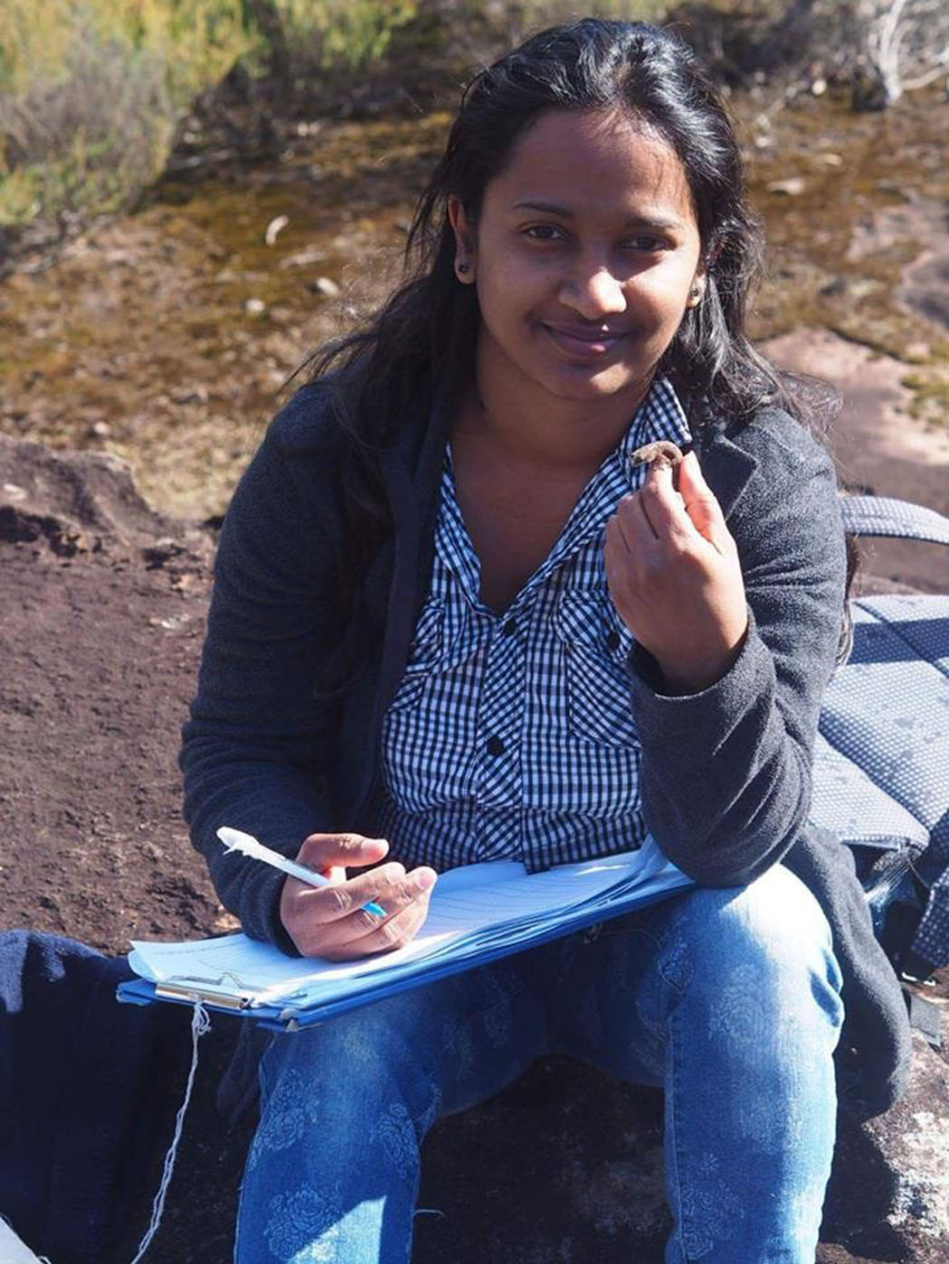


**Theja Abayarathna**

**What is your scientific background and the general focus of your lab?**

I started my research career as a student at the University of Sri Jayewardenepura, Sri Lanka. I love nature and started to explore the world of reptiles during my undergraduate degree and master's. I started my PhD at the University of Technology Sydney on a scholarship and had an opportunity to study lizard ecology under the guidance of my supervisor, Associate Professor Jonathan Webb. Our lab has a strong focus on reptile ecology, conservation biology, thermal biology and habitat restoration. For my PhD project, I investigated whether developmental plasticity might buffer velvet gecko populations from high temperatures that occur during heatwaves. In this species, females lay their eggs in communal nests inside sun-exposed rock crevices, and nest temperatures are positively correlated with air temperatures. During heatwaves, nests get hotter, so I wanted to find out how higher incubation temperatures would affect the morphology and physiology of hatchlings. To do this, I incubated eggs in programmable incubators to mimic current nest temperatures and ‘future’ temperatures likely to occur during hot summers. I then measured the thermal tolerance of hatchlings, their running speed and morphology, and released them at my study sites.

**How would you explain the main findings of your paper to non-scientific family and friends?**

Heatwaves are common in south-east Australia, and they can have adverse impacts on wildlife. During heatwaves, the communal nests of velvet geckos get hotter, which could potentially affect the embryos developing inside eggs. In some animals, exposure to higher temperatures during embryonic development can enhance the ability of offspring to tolerate higher temperatures later in life. This sort of predictive adaptive response is thought to increase the likelihood that the resulting offspring will have traits that will help them to survive in future environments. To see if this phenomenon occurs in velvet geckos, we incubated eggs to mimic current nest temperatures and future temperatures likely to occur during hot summers. We found that hatchlings from the future incubation temperature treatment could tolerate heat better than hatchlings from the current incubation temperature treatment. However, this effect was small and did not persist into later life, so it probably won't help geckos cope with higher temperatures that occur during heatwaves. Paradoxically, the geckos incubated under higher future temperatures were less able to tolerate cold than hatchlings from the current incubation temperature treatment. So in future, hotter nests may produce offspring that are poorly equipped to cope with cold winters.

“In some animals, exposure to higher temperatures during embryonic development can enhance the ability of offspring to tolerate higher temperatures later in life.”

**What are the potential implications of these results for your field of research?**

Our results have several implications. First, even though hatchlings from higher incubation temperatures had slightly higher heat tolerance, this effect was very small (less than 0.3°C) and it did not persist into later life. This suggests that developmental plasticity won't produce adaptive shifts in heat tolerance, at least in this lizard species. Second, higher incubation temperatures produced hatchlings with poorer cold tolerance. Based on field studies on other lizard species, individuals with higher critical thermal minima may have lower survival rates, particularly during cold winters. So, if these effects are general responses, the counterintuitive result is that hotter nests may produce lizards that are less able to cope with colder winters.

**Figure:**
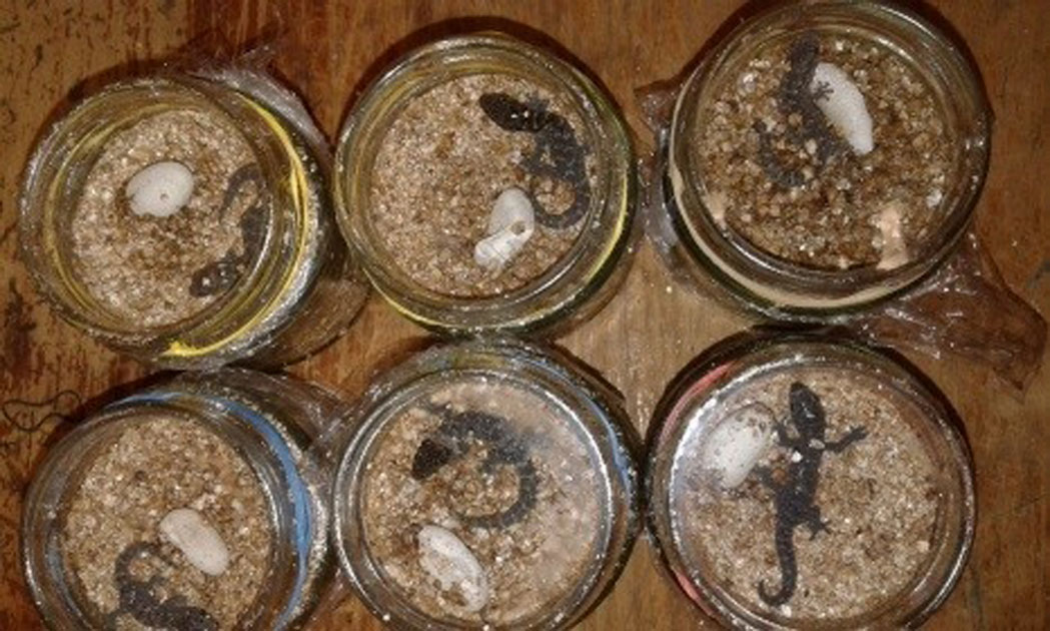
**Welcome to the world – new-born hatchling velvet geckos.**

**What has surprised you the most while conducting your research?**

I was surprised that higher incubation temperatures affected the cold tolerance of hatchling lizards. The critical thermal minima of current incubated lizards was 11.2°C, whereas the critical thermal minima of future incubated lizards was 14.1°C. This is a big difference, and I was not expecting to see this.

**What's next for you?**

I want to continue my research throughout my life. After I finish my PhD, I am planning to apply for a post-doc and carry out further research in this field.
